# Does Socio-Economic Status Have Different Impact on Fluid and Crystallized Abilities? Comparing Scores on Raven’s Progressive Matrices, Kaufman Assessment Battery for Children II Story Completion and Kilifi Naming Test Among Children in Ghana

**DOI:** 10.3389/fpsyg.2022.880005

**Published:** 2022-05-30

**Authors:** Adote Anum

**Affiliations:** Department of Psychology, University of Ghana, Accra, Ghana

**Keywords:** fluid intelligence, Ghana, coloured progressive matrices, Kilifi Naming Test, story completion task, crystallized intelligence

## Abstract

Current literature shows an association between intelligence and socio-cultural or socio-economic factors. The available evidence supports a stronger effect of exogenous factors on measures of crystalized intelligence than on fluid intelligence. Despite this, the sources of variability in fluid and crystalized intelligence have not been explored adequately in intelligence research. The purpose of this study was to compare performance on tests that measure fluid and crystallized intelligence among children selected from public and private schools in Ghana. We tested the assumption that socio-economic status (SES) will have a stronger effect on tests that measure crystallized intelligence than on fluid intelligence. We selected 185 children between 6 and 12 years from private and public schools, and used inclusion in a private or public school as a proxy for SES. We administered the Raven’s Coloured Progressive Matrices (RCPM), a fluid intelligence test, the KABC II story completion subtest as a measure of inductive reasoning and crystallized intelligence and the Kilifi Naming Test, a verbal ability measure designed to minimize the effect of school on vocabulary. The results showed age-related improvement in scores on all three tests with effect sizes ranging from 0.42 to 0.52. We also found significant effect for type of school on all the tests with effect sizes ranging from 0.37 to 0.66. The results also showed an increasing disparity in performance on the tests favoring children selected from private schools. These suggest that fluid and crystalized intelligence are affected by socioeconomic factors. The results also showed that SES factors tend to affect crystallized ability more than it affects fluid ability. The results are discussed in the context of differences in socioeconomic resources available to children such as quality of education in low- and middle-income countries.

## Introduction

Children’s performance on intelligence and cognitive ability tests is influenced by a variety of factors, most importantly genetics, maturation, socio-economic characteristics, health, nutrition, testing system used, and test characteristics (Plomin and Petrill, 1997; van de Vijver and Tanzer, 2004; Fanjiang and Kleinman, 2007). In low- and middle-income countries or in non-Western contexts, socio-economic influences are more pronounced. First, there are large disparities in socio-economic characteristics in non-Western countries. Socio-economic status (SES) has an effect on parenting (Hoff-Ginsberg and Tardif, 1995), nutrition (McCoy et al., 2015), and access to quality education, all of which have significant effect on cognitive and intellectual development (Sigman and Whaley, 1998; Hanscombe et al., 2012; Zamroziewicz et al., 2017). Second, most intelligence tests are developed in Western countries and could be biased when used in non-Western contexts. The use of Western instruments in non-Western contexts may lead to inaccurate assessment and labeling (van de Vijver and Tanzer, 2004; Verney et al., 2005). To eliminate or minimize these biases, tests are validated and frequently re-standardized in local populations. Standardization changes norms, but does not remove the effects on test scores caused by other factors such as parents’ socio-economic status and school-related characteristics such as quality of school and teaching. These vary significantly enough in developing countries to affect test scores. The effects of these SES related factors are more pronounced on crystallized intelligence tests and to a lesser extent on fluid intelligence tests which require abilities that are largely considered innate (or biological) and not easily affected by external factors (Brody, 1997; Désert et al., 2009). Research on intelligence in sub-Saharan African countries, including Ghana, have consistently found lower scores on both standardized fluid and crystallized intelligence tests. Other evidence, however, suggests that when SES is considered, children with high SES backgrounds obtain standard scores comparable to Western published norms (e.g., Anum, 2014). This raises the question whether single normative data adequately describe intelligence among children in sub-Saharan Africa and if SES has similar effects on both fluid and crystallized intelligence. We therefore investigated the extent that scores on tests that measure fluid and crystallized intelligence are affected by SES among a sample of children categorized into high and low SES groups.

### Fluid and Crystallized Intelligence

The distinction between fluid and crystallized intelligence is the outcome of almost 100 years of accumulated research (Carroll, 1993; Kent, 2017). Fluid intelligence (Gf) is defined as the ability to solve problems through reasoning and according to Carroll (1993) is central to all intellectual ability (Colom and García-López, 2002). Typically, tests designed to measure fluid intelligence are designed to be culture-fair; meaning performance should be less hindered by external characteristics such as education or culture. Performance on fluid intelligence tests is therefore deemed to depend largely on innate ability or genetics and less on socio-cultural characteristics. There is increasing research, however, that has shown that fluid intelligence tests are not only influenced by external factors but can also be improved in individual development (Sternberg, 2008). The Raven’s Progressive Matrices is one of the most commonly used measures of fluid intelligence. Crystallized intelligence (Gc) is a knowledge-based ability that is influenced by education and acculturation. Performance on tests that measure crystallized intelligence is therefore influenced greatly by factors such as culture, parenting practices, schooling and quality of school and teaching. Tests that measure verbal ability and/or the knowledge components of most standardized intelligence tests typically measure crystallized intelligence (Schipolowski et al., 2014). Different tests therefore measure fluid and crystallized abilities, respectively.

Research in sub-Saharan Africa, especially in South Africa and Ghana have shown that test scores on fluid intelligence are lower when compared on United Kingdom and United States published norms (Rushton et al., 2003; Brouwers et al., 2009; Wicherts et al., 2010; Nijenhuis and van den Hoek, 2016). The consistently low intelligence test scores on standardized tests in sub-Saharan Africa is attributed to innate deficit in intellectual ability and not test or cultural biases (see for example, Rindermann, 2013; Nijenhuis and van den Hoek, 2016). This is however challenged by other studies that have shown that socio-economic or socio-cultural factors are associated with differences in fluid intelligence in sub-Saharan African countries (Knoetze et al., 2005; Brown and Day, 2006; Anum, 2014). These findings created a need to address the question about the extent to which socio-economic factors can influence intelligence test scores and if the observed differences are consistent for both fluid and crystallized intelligence.

To answer these questions, it will be necessary to compare intelligence test scores of children from high and low socio-economic background on tests that measure fluid and crystalized abilities. In this study, we examined differences in performances on three tests that are assumed to measure fluid and crystallized abilities.

### Socio-Economic Status and Intelligence

Richer and more heavily resourced countries provide adequate educational infrastructure and social policies that ensure more uniform access to high-quality education and health care to most children. This creates a more supportive environment for most children which then leads to positive outcomes on children’s cognitive development. In low- and middle-income countries, resource scarcity and inequitable allocation create large disparities. Access to quality education is normally limited to individuals in middle to high socio-economic groups and individuals living in more urbanized communities.

Socio-cultural and socio-economic factors encompass a number of inter-related variables that include but are not limited to school attendance, school quality, educational attainment, home and schooling experiences and parental characteristics like parents’ education and income (Gonzales and Roll, 1985; Olazaran et al., 1996; Shuttleworth-Edwards et al., 2004). In Africa, research from Libya (Al-Shahomee and Lynn, 2010; Bakhiet and Lynn, 2015), Egypt (Abdel-Khalek, 1988), Ghana (Anum, 2014), Kenya (Costenbader and Ngari, 2001), and South Africa (Knoetze et al., 2005; Lynn and Meisenberg, 2010a) have established that test scores and IQ equivalents are significantly lower than expected, sometimes by more than 1 standard deviation. These findings have been the basis for the debate of whether lower scores on intelligence tests among sub-Saharan African children are due to genetics or to the environment within with children grow (Rindermann, 2013). We focus on the influence of SES-environment—in this paper.

The Raven’s Progressive Matrices, commonly used to measure fluid intelligence, is considered a culture-fair test. In spite of being culture-fair, a number of studies have reported that scores on the Raven’s Progressive Matrices are influenced by formal education (Brouwers et al., 2009; Bakhiet and Lynn, 2015). Research on crystallized intelligence have shown significant associations between test scores and socio-economic characteristics. For example, in Sudan, data from the WISC showed that IQ equivalents were significantly lower in the most southerly region compared to areas in the North of Sudan. The southern portions are the least developed regions of Sudan (Bakhiet and Lynn, 2015). These studies contribute to increasing evidence that associate IQ with socio-economic characteristics (Lynn and Meisenberg, 2010b; Meisenberg and Lynn, 2011; Lynn and Vanhanen, 2012; Bakhiet and Lynn, 2015).

The effect of environmental factors on crystallized intelligence is established (see for example, Rindermann et al., 2010; Schroeders et al., 2016). The sources of within-group variability in fluid general intelligence have not been systematically investigated in low- and middle-income countries where differences in abilities are bound to be greatest because of disparities in income and access to quality education and other factors that affect cognitive development of children. One reason is that intelligence researchers work under the assumption that fluid intelligence tests measure abilities that are innate and therefore not affected by external factors.

Cross-cultural research on cognitive ability use aggregated samples—groups from different socio-economic backgrounds. In sub-Saharan Africa, resources that likely influence cognitive development are not equitably distributed across SES groups and across the rural–urban divide. Therefore, test scores based on aggregated means do not take into account the variability in SES related factors. Studies in which SES groups are disaggregated tend to show differences in the distributions of scores; higher mean scores for middle to high SES groups than for low SES groups (Owen, 1992; Anum, 2014; Mitchell et al., 2018; Poh et al., 2019). In Ghana, for example, Anum (2014) found that children in medium to high socio-economic groups obtained higher test scores than those in lower socio-economic groups. Among children in the higher SES group, the scores on the Raven’s Coloured Progressive Matrices of children were comparable to published United Kingdom norms. This finding contributes to the evidence supporting the effects of socio-economic factors and therefore has to be considered when interpreting fluid intelligence test scores.

### Current Study

Intelligence and developmental theories suggest a systematic growth in mental ability from infancy to maturity (Jensen, 1980). Among young children, there is an expected rapid increase in test scores from infancy to adolescence. The rate of improvement, however, has been shown to vary depending on a variety of factors. Some evidence for the influence of socio-economic and cultural factors on intelligence comes from research on differences between black and whites in United States. For example, multiple studies in the United States have shown that black children scored significantly lower than white children, a difference that diminished when family and household characteristics are statistically controlled (e.g., Brooks-Gunn et al., 1996; Duncan and Magnuson, 2005).

Despite the known associations between intelligence and SES, the influence of socio-economic characteristics on intelligence test scores is not investigated often in sub-Saharan Africa. The purpose of this study was to examine this association on fluid and crystallized intelligence, using the Raven’s Coloured Progressive Matrices, the KABC II story completion subtest, and the Kilifi Naming Test (KNT) in a sample of children in public and private schools in Accra, Ghana.

The major objective was to test the extent to which SES influences score on the Raven’s Progressive Matrices, a fluid intelligence test, KABC II Story completion test and KNT. The KABC subtest and KNT are tasks that measure crystallized intelligence. It was predicted that SES effect will be stronger on tests that measure crystallized intelligence than on the fluid intelligence test. The prediction was that, (1) there will be age-related increases on the fluid and crystallized intelligence tests, and (2) the age-related increases will be higher among high SES group than among low SES group.

The Raven’s Progressive Matrices is a nonverbal measure of fluid intelligence, requiring ability not influenced by external factors. The KABC II story completion is a measure of induction, visualization, sequential reasoning, and general information (Kaufman and Kaufman, 2004). Compared to the RCPM, the stimuli are highly culturally sensitive and therefore favors individuals exposed to foreign concepts. The KNT is a verbal ability test on which children are required to name common objects from the environment either in English or in the child’s native language. Naming the objects in a language that is most convenient to the child minimizes the limitation that school attendance or school quality might place on children’s performance on the test.

## Materials and Methods

### Participants

Three schools, two public and one private, were purposively selected from one district in the Eastern part of Accra for this study. The schools had children from first to sixth grade. We used type of school attended (private or public), as a proxy for SES (Agbozo et al., 2016; Gaddah et al., 2016). Children enrolled in both public and private school facilities create a ready pool of participants classified by SES. Private schools typically attract children from middle to upper income groups. Parents of children in public schools in Ghana are generally of low-income. Tuition and other related costs in the public schools are borne by the government (Bidwell et al., 2014).

A systematic sampling approach was used in the private school where class sizes ranged from 25 to 30 children. We selected about 30% of children in each class. We did not follow this selection method in the public schools where it is typical for children to be slightly older and therefore a random selection might lead to oversampling older children in each class. Children are older in public schools because it is common for them to drop out and re-enroll later. Average age for first grade in Ghana is 6 years.

### Measures

Three tests were selected to measure general fluid intelligence, conceptual reasoning, and expressive verbal ability.

#### Raven’s Coloured Progressive Matrices

This is the children’s version of the Raven’s Progressive Matrices which was designed to measure general fluid intelligence (Raven, 2000). The Raven’s Progressive Matrices is described as the best measure of general intelligence. The coloured version was designed for children aged 5–11 years, the elderly, and intellectually impaired individuals. It has three sets; the first two are coloured and the third is in black and white. Each set has 12 items. It is a nonverbal test and children are required to select a response from an array that correctly completes a pattern.

#### Kaufman Assessment Battery for Children II Story Completion

The Story completion is a subtest of the KABC II is a measure of crystallized intelligence. It measures pattern recognition, reasoning, and planning. The child is shown a row of pictures that tell a story with some of the pictures missing. The child is required to select the missing pieces from a given array and has to place the pictures in their correct locations. This test is highly sensitive to external factors such as education and exposure and therefore performance is more likely to be affected by school attendance and social background.

#### Kilifi Naming Test

The Kilifi Naming Test was developed in Kenya to measure expressive vocabulary among children in a rural setting (Kitsao-Wekulo et al., 2019). The child must provide labels for a selection of pictures presented in black and white with or without cues. The child is expected to provide a name for the object in the picture either in English or in the local language within a specified time. On a typical item, a child is shown the picture of baobab tree and the child is required to provide the name either in English or in his/her local dialect.

### Procedure

Two research assistants who had obtained BA in Psychology were trained by a trained clinical psychologist. Training involved a combination of theory and practice sessions of test administration spanning 1 week which also included practice sessions on children in another school (not selected for this study). The study received permission from Ethics Committee for the Humanities of the University of Ghana (Ethics Number: ECH 021/19–20). We also obtained permission and consent from the respective schools’ authorities. Children who were selected gave verbal assent before participation.

Testing was done in the Schools’ library. The public schools were in close proximity to each other and there testing was done in one school’s library. Five children were tested every day. The administration was divided into two test sessions. The first session included collecting background information, including taking biometric information such as weight, height, and mid-upper arm circumference, and then tested on story completion. This was followed by a 15-min break and then tested on RCPM and KNT. The entire assessment lasted for 90 min for each child.

### Analytic Plan

Three analytic strategies were used. First, descriptive statistics were examined to determine the average scores on each test. Second, we tested responsiveness of the tests to changes in age and school type. The intention was to see if there were significant changes in performance on tests caused by increases in age. Third, we calculated the variance of sex, age, and school type on each of the tests.

## Results

In total, 185 children, with ages ranging between 6 and 12 years were tested from three schools, two public schools and one private school. The summary of sample and age characteristics is presented in Table 1. The children were grouped into six age groups: 6–7, 7–8, 8–9, 9–10, 10–11, and 11–12. Females consistently outnumbered males (female = 54%) across all age groups except for 11–12 years.

**Table 1:** Distribution of the sample in the study by age and gender.

Age group (years)	Numbers and frequencies (%)^a^					
	Combined		Public schools		Private schools	
	Males	Females	Males	Females	Males	Females
6–7	16 (47.1)	18 (52.9)	11 (42.3)	15 (57.7)	5 (62.5)	3 (37.5)
7–8	11 (37.9)	18 (62.1)	6 (30.0)	14 (70.0)	5 (55.6)	4 (44.4)
8–9	10 (43.5)	13 (56.5)	4 (28.6)	10 (71.4)	6 (66.7)	3 (33.3)
9–10	9 (39.1)	14 (60.9)	3 (23.1)	10 (76.9)	6 (60.0)	4 (40.0)
10–11	15 (44.1)	19 (55.9)	11 (50.0)	11 (50.0)	4 (33.3)	8 (66.7)
11–12	24 (58.8)	17 (41.5)	16 (55.2)	13 (44.8)	8 (66.7)	4 (33.3)
Total	85 (46.2)	100 (53.8)	51 (41.1)	73 (58.9)	34 (56.7)	26 (43.3)

a *Frequencies in parentheses*.

Normality of scores reported on Table 2 was based on skewness value between +2 and −2. All the test scores were evaluated as normal. The results showed that on all tests for both public and private schools, scores ranged from lowest toward the maximum possible. Scores for children in the private school were higher on all three tests, substantially higher on RCPM and KABC II story completion. The mean scores for each age group are presented on Table 3. The means for all tests followed the expected increasing trend associated with increase in age.

**Table 2:** Sample variance for tests.

Combined	*N*	Max possible	Mean	SD	Min	Max
RCPM	184	36	19.26	7.33	2	36
KABC II story completion	185	47	10.11	8.88	0	36
Kilifi Naming Test	185	122	47.82	19.93	9	94
Public						
RCPM^a^	125	36	16.35	5.72	2	32
KABC^b^ II story completion	125	47	5.91	3.84	0	21
Kilifi Naming Test	125	122	40.71	15.30	9	76
Private						
RCPM	60	36	25.28	6.59	11	36
KABC II story completion	60	47	18.88	9.96	1	36
Kilifi Naming Test	60	122	62.62	20.42	27	94

a *RCPM, Raven’s coloured progressive matrices*.

b *KABC, Kaufman assessment battery*.

**Table 3:** Mean and standard deviation of tests for each age group.

Age groups	Mean (SD)								
RCPM^a^			KABC^b^ II story completion			Kilifi Naming Test		
	**Combined**	**Public**	**Private**	**Combined**	**Public**	**Private**	**Combined**	**Public**	**Private**
6–7 years	12.97(3.12)	11.92(2.22)	16.25(3.37)	3.65(2.33)	3.04(1.61)	5.63(3.25)	27.21(10.30)	24.38(9.94)	36.38(4.60)
7–8 years	15.28(4.74)	13.05(1.99)	20.22(5.43)	7.00(5.39)	5.15(2.58)	11.11(7.62)	37.76(12.39)	36.45(13.52)	40.67(9.46)
8–9 years	19.52(5.15)	17.14(4.69)	23.22(3.46)	9.00(5.60)	5.36(2.24)	14.67(4.33)	46.61(13.84)	42.57(11.95)	52.90(14.90)
9–10 years	22.52(7.53)	17.62(3.80)	28.90(6.28)	13.43(10.23)	5.92(1.71)	24.30(6.32)	59.04(18.90)	46.85(15.44)	74.90(7.20)
10–11 years	23.00(7.30)	17.90(4.98)	29.92(3.12)	14.29(10.00)	7.91(4.19)	26.00(5.98)	60.50(16.88)	49.82(9.00)	75.00(13.98)
11–12 years	23.53(6.36)	21.61(5.78)	30.17(2.98)	15.07(9.36)	8.52(4.51)	27.83(6.06)	61.00(18.15)	50.65(11.46)	82.25(9.39)

a *Raven’s coloured progressive matrices*.

b *Kaufman assessment battery*.

Between-group differences. The effects of two independent factors, age and SES were computed using between-group analyzes of variance on all three measures. As indicated previously, age was recategorized into a 12-month band. Type of school, public and private were used as a proxy for SES. Summary of between-group analysis is presented in Table 4.

**Table 4:** Effects of age and SES (type of school) on RCPM^a^, KABC^b^ II story completion, and Kilifi Naming Test.

RCPM	*df*	*F*	*p*	Effect size (*η*^2^)
Age groups	5	31.61	0.001	0.48
SES (type of school)	1	146.28	0.001	0.46
Age * SES	5	3.20	0.001	0.09
*KABC II story completion*				
Age groups	5	46.29	0.001	0.57
SES (type of school)	1	326.20	0.001	0.66
Age * SES	5	19.51	0.001	0.36
*Kilifi Naming Test*				
Age groups	5	43.62	0.001	0.60
SES (type of school)	1	101.08	0.001	0.37
Age * SES	5	6.31	0.001	0.16

a *RCPM, Raven’s coloured progressive matrices*.

b *Kaufman assessment battery II story completion*.

### Raven’s Coloured Progressive Matrices

**Figure 1:**
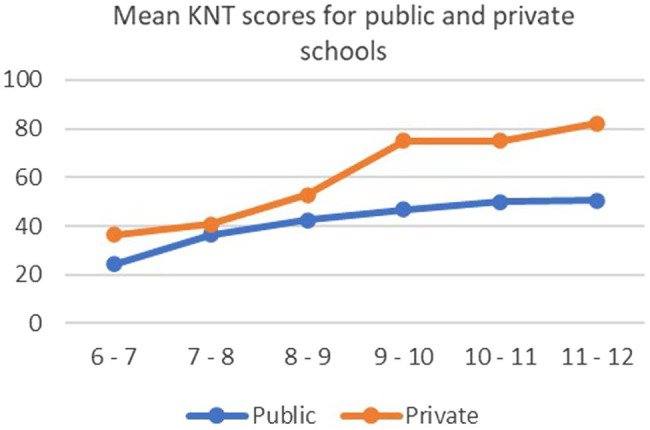
Graph showing mean scores on RCPM for age groups by public and private schools.

There was a strong effect for age, that is, higher mean scores were associated with higher age groups. This is a normal expectation among children. There was also a significant effect for SES on this test. For both public and private schools, the age-related changes were linear (Figure 1). There was also a significant interaction between age and SES. The results showed the difference between the mean scores increase slightly between the public and private schools among 9 to 11 years, in favor of children in private schools.

### Kaufman Assessment Battery for Children II Story Completion

**Figure 2:**
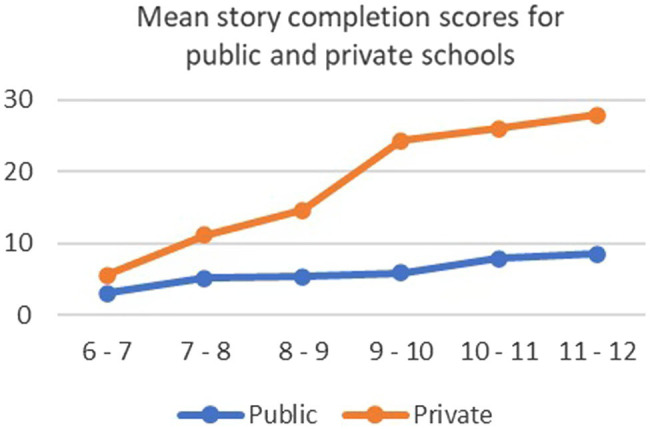
Graph showing mean scores on KABC II story completion test for age groups by public and private schools.

There was also a statistically significant effect for age. Again, as expected, older children improved in mean scores in both SES groups. There was a very strong effect of SES on the test, with children in private schools having increasingly higher means as age increased. This was confirmed in the results for the interaction effect which showed that the interaction between age and SES was significant, showing an increasing gap between the two SES groups at higher than lower ages. It was expected that children in private schools will score higher than children in public schools. Examination of the means on Table 3 and effect sizes on Table 4 showed the improvement in scores as age increased was greater on story completion than on the Raven’s Progressive Matrices and KNT (Figure 2).

### Kilifi Naming Test

**Figure 3:**
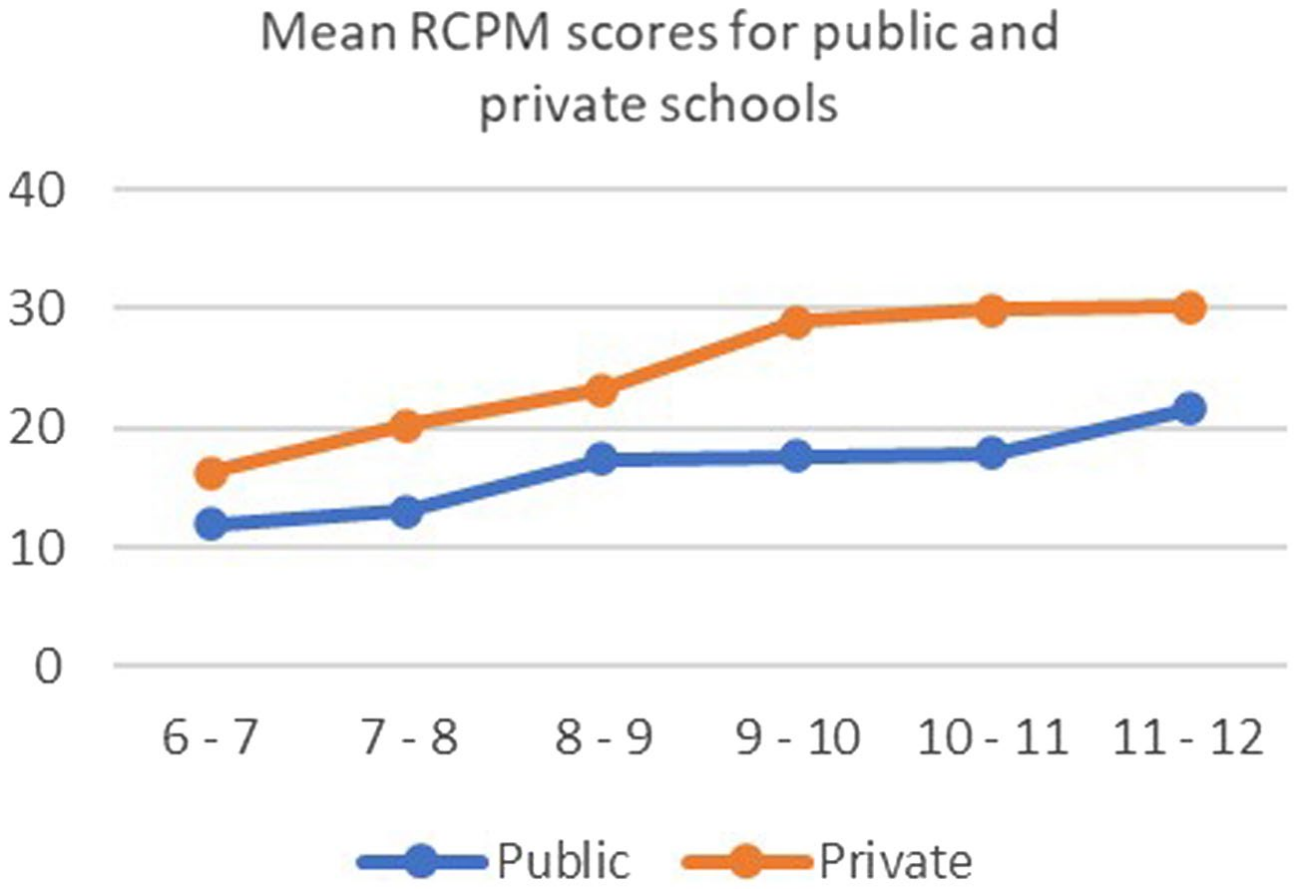
Graph showing mean scores on KNT test for age groups by public and private schools.

Consistent with result on the RCPM and KABC II story completion, there was a significant increase in scores on KNT with age. There was also a significant effect for SES; children in the private school had a higher mean score than children in public school. The age-related change in score was less steep among children in public school than for children in private school (Figure 3). This is confirmed in the test for interaction effect which showed a significant interaction between age groups and type of school. It was found that in all three tests the mean scores were nearly similar at younger ages. The gap between the two SES groups increases with increase in age.

## Discussion

This study was carried out to compare performance on Raven’s Coloured Progressive Matrices, the KABC II subtest story completion, and the Kilifi Naming Test as measures of fluid and non-fluid intelligence. We found age associated changes on all three measures. We also found that children in private schools had higher scores on all tests, an indication that socio-economic factors accounted for scores on the cognitive measures.

Raven’s Coloured Progressive Matrices measure general fluid intelligence, an ability that is generally considered to be more innate and less likely to be influenced by exogenous factors. The SES-related result from this study is yet another evidence from low- and middle-income countries that show that fluid ability is influenced by exogenous factors such as school quality, parents’ characteristics and a host of environmental factors (Rindermann et al., 2010, 2015; Flores-Mendoza et al., 2018; Peng et al., 2019). The degree of influence however is lower than for crystallized intelligence. In countries like Ghana, where SES differences tend to have significant impact on access to quality education, health support, and other infrastructure that, researchers must reexamine sources of variability within populations of varying socio-economic and socio-cultural characteristics. Children from the private school across all age groups consistently obtained superior scores, sometimes by more than 1 standard deviation. We explain the difference between the two groups by examining the external factors that distinguish the groups such as the quality of school and other socio-economic factors.

Public schools in Ghana are bedeviled by poor infrastructure, lack of adequate and appropriate teaching aids, high student-teacher ratios, and poor teacher supervision (Ampiah, 2008; Adu-Agyem and Osei-Poku, 2012). These are factors that affect quality of education and therefore have impact on cognitive development and achievement. Of the two public schools used in the study, both had no functional library. Class sizes ranged from 50 to 70 children per class. These are typical of public schools in Ghana (Opoku-Amankwa, 2010). Since these affect cognitive development and intelligence test scores, IQ estimates, based on fluid intelligence test scores should be interpreted with caution. This means, test users should be mindful of the influence of the child’s background and experiences when selecting and interpreting test scores.

The results for KABC II story completion further strengthened the association between SES and intelligence. The KABC II has not been validated in Ghana and therefore there are no normative data against which we can compare the scores obtained in this study. When compared to the published norms the performance of children in the private school was closer to the average standard score of 10, much higher than the scores of children in public schools (Appendix). Among children in the higher SES group, age effect was stronger after 9 years. This finding suggests older children gain from cumulative impact of better school quality which reflected in a greater improvement on story completion. This is similar to an earlier finding by Tucker-Drob and Bates (2016) who reported that SES effects tend to suppress innate ability when comparing high and low SES groups on school achievement (Tucker-Drob and Bates, 2016).

Scores on the KNT showed a linear age trend and significant type of SES effect. The SES effect contradicted our prediction which was based on the assumption that requiring children to name objects in their local language should eliminate or minimize school or SES effect. It appears however that performance on the KNT reflected a fundamental language ability. Language development is accounted for by several factors such as parental characteristics, education, school characteristics (Raviv et al., 2004; Pungello et al., 2009).

SES related differences in fluid and crystallized abilities are not isolated findings (Raven, 2000; van Tuijl and Leseman, 2007; Vista and Grantham, 2010; Anum, 2014; Rindermann et al., 2015; Flores-Mendoza et al., 2018; Peng et al., 2019). For example, Rindermann et al. (2015), when comparing Costa Rican and Austrian children, found a greater difference among secondary school children than among preschoolers. They also found larger differences on crystallized measures than on a fluid measure.

These and similar results suggest that low SES places children at a disadvantage which manifests in low scores on cognitive ability tests. SES is made of a multiplicity of factors such as parents’ education, occupation, income, school attendance, and adequate home supervision which form a foundation for children’s cognitive development. Wolf and McCoy (2019) for example, found in a study in Ghana that school-readiness was a direct function of SES among other factors that include number of books in the house (Wolf and McCoy, 2019). In an earlier study, Ritchie et al. (2015) also found that there is a direct association between education and cognitive test scores, a relationship that is not mediated by general intelligence (Ritchie et al., 2015).

Children who come from low SES groups in low- and middle-income countries are disproportionately exposed to environmental risks (such as poor school infrastructure and pollution) and have unfavorable parent characteristics such as low education, low income and low parental involvement, all of which adversely affect cognitive development and lead to low educational attainment (McCoy et al., 2016).

Findings from this study bring further clarity to the issue of the extent of influence of exogenous factors on the Raven’s matrices measure—an ability intended to be measured without an influence by exogenous factors. The findings also show that rate of age-related increase in intelligence test scores is higher on crystallized than on fluid intelligence tests and this variability is likely shaped more by the socio-economic characteristics. Previous studies have not shown this distinction. Further, the findings contribute to theory building by providing needed evidence that explain the effect of SES on intelligence in contexts that have large socio-economic disparities.

### Conclusion

We found all the tests in this study to be sensitive to changes in age for children in both high and low SES background. The rate of age-related change however was not the same for both SES groups. The study adds to existing literature that show fluid intelligence (and the Raven’s Progressive Matrices) are influenced by SES factors such as education and school quality. Often times, the influence of SES on fluid intelligence is ignored. Findings from this study and previous findings should remind researchers, particularly in low- and middle-income countries to examine more closely the sources of variability on fluid intelligence test scores. Because of the multiplicity of factors that can explain differences in intelligence in low- and middle-income countries, of which SES is very important, we must begin to examine if having unitary normative data on intelligence tests adequately describes performance for all children in sub-Saharan Africa.

Children in sub-Saharan Africa consistently score low on standardized tests that measure both fluid and crystallized intelligence. The findings from this study should begin the quest to explore alternative explanations for the low scores and possibly develop more culturally relevant assessment methods or instruments.

### Limitations

There are some limitations that must be considered when interpreting the results from this study. First, the study was done in the capital city of the Ghana where facilities are relatively better than in rural areas. We did not have the benefit of a probability selection of schools that may be more representative of schools across the country. Results therefore cannot be easily generalized to the rest of the country. Secondly, the sample size is relatively small. Studies with reasonably large sample sizes and using random selection methods do not only ensure representativeness of sample but also provide stability in scores for all age groups. This is a cross-sectional study in which we collected data from different cohorts at the same time. A longitudinal study that provides continuous evidence of lower scores for the same children across time will better explain the observed phenomenon. Thirdly, the design of this study did not allow for manipulated independent variables. The use of fixed factors—SES (school type) does not allow for conclusions about causal relations even with very high effect sizes. There are inherent factors such as genetic and parental factors that potentially affect test scores which are not accounted for in this study. Heritability studies have shown an increasing genetic impact with age (e.g., Johnson, 2010). In other words, when children get older, the influence of hereditary on cognitive abilities appear to be stronger. Higher parental involvement such as in helping children with schoolwork also has a strong impact on cognitive development and school achievement. Fourthly, we did not directly measure parent characteristics like education, parental involvement, and parenting styles, factors that affect cognitive development and achievement. We can therefore only speculate about these from one dimension, attendance in either public or private schools. Finally, the scope of this study is relatively small using only three cognitive measures. There are several cognitive domains omitted in this study. For example, memory, attention, academic achievement, and other culturally specific abilities when present provide a more comprehensive examination of the issues explored. In the future, researchers should consider using a larger sample, measuring abilities in several cognitive domains to provide a broader perspective of the issues we have examined in this study.

Raven’s Progressive Matrices is a commonly used measure when researchers are interested in cross-cultural comparisons. Using Raven’s Progressive Matrices does not insulate researchers and their findings from the influence of SES on intelligence and cognitive ability. To disentangle the effect of social and external characteristics from intelligence tests would require a comparison of ability on fluid and non-fluid ability tests, among children from different socio-economic backgrounds.

## Data Availability Statement

The raw data supporting the conclusions of this article will be made available by the authors, without undue reservation.

## Ethics Statement

The studies involving human participants were reviewed and approved by Ethic Committee for the Humanities of the University of Ghana (Ethics Number: ECH 021/19–20). Written informed consent from the participants’ legal guardian/next of kin was not required to participate in this study in accordance with the national legislation and the institutional requirements.

## Author Contributions

AA conceptualized, drafted, and reviewed the manuscript.

## Conflict of Interest

The author declares that the research was conducted in the absence of any commercial or financial relationships that could be construed as a potential conflict of interest.

## Publisher’s Note

All claims expressed in this article are solely those of the authors and do not necessarily represent those of their affiliated organizations, or those of the publisher, the editors and the reviewers. Any product that may be evaluated in this article, or claim that may be made by its manufacturer, is not guaranteed or endorsed by the publisher.

## Figures and Tables

**Table:** 

Age groups (years)		Mean (SD)	
	Combined	Public	Private
6–7	6.35(2.41)	5.40(1.92)	8.13(2.30)
7–8	5.64(2.92)	4.14(1.83)	8.25(2.66)
8–9	5.82(3.05)	3.25(1.83)	8.11(1.76)
9–10	6.68(3.68)	3.22(0.44)	9.88(2.04)
10–11	6.37(3.97)	3.08(2.02)	9.67(2.27)
11–12	5.37(3.44)	3.42(1.79)	9.63(1.91)

## Appendix

Mean standard scores of Kaufman Assessment Battery II Story completion.
